# Acute Scrotal Edema as the Initial Presentation of Inferior Vena Cava Stenosis With Secondary Thrombosis After Right Hepatectomy: A Case Report

**DOI:** 10.7759/cureus.114798

**Published:** 2026-08-19

**Authors:** Melis Geyik, Eray Huroglu, Servet Karagul, Cuneyt Kayaalp

**Affiliations:** 1 General Surgery, Atlas Üniversitesi, Istanbul, TUR

**Keywords:** cholangiocarcinoma, hepatectomy, inferior vena cava thrombosis, scrotal edema, venous reconstruction

## Abstract

Inferior vena cava (IVC) thrombosis is a rare complication after major hepatectomy. Delayed diagnosis may result in venous congestion, acute kidney injury, and other serious complications. A 57-year-old man underwent right hepatectomy for perihilar cholangiocarcinoma. On the second postoperative day, he developed oliguria, worsening renal function, and sudden scrotal swelling. Doppler ultrasonography showed preserved testicular perfusion with diffuse scrotal edema. CT angiography demonstrated partial thrombosis of the IVC just below the hepatic venous confluence, causing marked narrowing of venous flow. Emergency reoperation revealed mechanical stenosis of the IVC adjacent to the right hepatic vein stump. Thrombectomy was performed, and the narrowed segment was enlarged using an autologous parietal peritoneal patch. Renal function improved after surgery, urine output increased, and the scrotal edema resolved completely during follow-up. Sudden scrotal edema after hepatectomy may be the first clinical sign of IVC obstruction. Early imaging and prompt surgical treatment can prevent permanent renal and vascular complications.

## Introduction

Major hepatectomy is associated with several well-known postoperative complications, including bleeding, bile leakage, liver failure, and infection. Vascular complications occur less frequently but may have serious consequences. Among them, inferior vena cava (IVC) stenosis and thrombosis are particularly uncommon and may impair hepatic and renal venous drainage if diagnosis is delayed [1]. Patients undergoing liver resection are at increased risk of postoperative venous thromboembolism. Ejaz et al. reported an incidence of approximately 4-5% after hepatectomy, with prolonged operative time and hospital stay identified as important risk factors [2]. In most patients, however, isolated IVC thrombosis is not related to hypercoagulability alone. Mechanical narrowing of the vessel may also contribute to thrombus formation [3]. In such patients, the primary lesion is a mechanical stenosis, and the intraluminal thrombus is a secondary phenomenon. This distinction is clinically important, because anticoagulation or thrombectomy alone does not correct the underlying narrowing, and definitive treatment requires enlargement of the stenotic segment.

Scrotal edema is usually associated with systemic fluid overload or local scrotal disease. As an isolated manifestation of acute IVC obstruction after hepatectomy, it has rarely been described. We present a patient in whom sudden scrotal edema was the first clinical finding that led to the diagnosis of postoperative IVC stenosis with secondary thrombosis.

## Case presentation

A 57-year-old man was admitted with progressive jaundice and right upper quadrant pain. He had no significant medical history other than an open cholecystectomy performed in 1996. His father had died of lung cancer. Physical examination showed jaundice of the skin and sclera. A percutaneous transhepatic biliary drainage (PTBD) catheter had been inserted before referral. Laboratory tests demonstrated a total bilirubin level of 4.09 mg/dL with a cholestatic pattern of liver enzymes. Mild anemia (hemoglobin 11.2 g/dL) and mildly elevated aminotransferase levels (AST 64 U/L, ALT 74 U/L) were present. Viral hepatitis and HIV serology were negative. Renal function and coagulation tests were within normal limits (Table 1).

**Table 1:** Selected laboratory findings during the clinical course Abbreviations: POD, postoperative day; AST, aspartate aminotransferase; ALT, alanine aminotransferase; eGFR, estimated glomerular filtration rate; BUN, blood urea nitrogen; PT, prothrombin time; INR, international normalized ratio; aPTT, activated partial thromboplastin time.

Parameter	Preoperative	POD 1	POD 2	Pre-discharge	Reference Values
Hemoglobin (g/dL)	11.2	12.8	10.8	11.5	13.2-17.3
Platelet (10^3^/uL)	192	225	267	86	150-400
Sodium (mmol/L)	141	136	134	136	135-145
Potassium (mmol/L)	4.0	5.0	3.8	3.5	3.60-4.80
Total bilirubin (mg/dL)	4.09	3.7	2.39	1.43	0.1-0.7
Direct bilirubin (mg/dL)	3.00	2.84	1.73	0.89	0-0.5
Lactate (mmol/L)	-	1.06	1.29	-	0.00-2.00
Albumin (g/dL)	3.5	3.3	3.0	3.4	3.4-5.0
AST (U/L)	64	283	116	34	5-34
ALT (U/L)	74	222	155	19	0-55
Creatinine (mg/dL)	0.81	1.13	2.84	0.50	0.70-1.12
eGFR (mL/min/1.73 m²)	97.73	71.41	23.49	119.31	≥90
BUN (mg/dL)	19.7	26.4	52.2	6.6	6-20
PT (sec)	14.5	13.9	15.3	13.6	10-14
INR	1.24	1.19	1.31	1.16	0.8-1.2
aPTT (sec)	24.6	22.9	33.2	29.9	22.1-30.2

**Figure 1:**
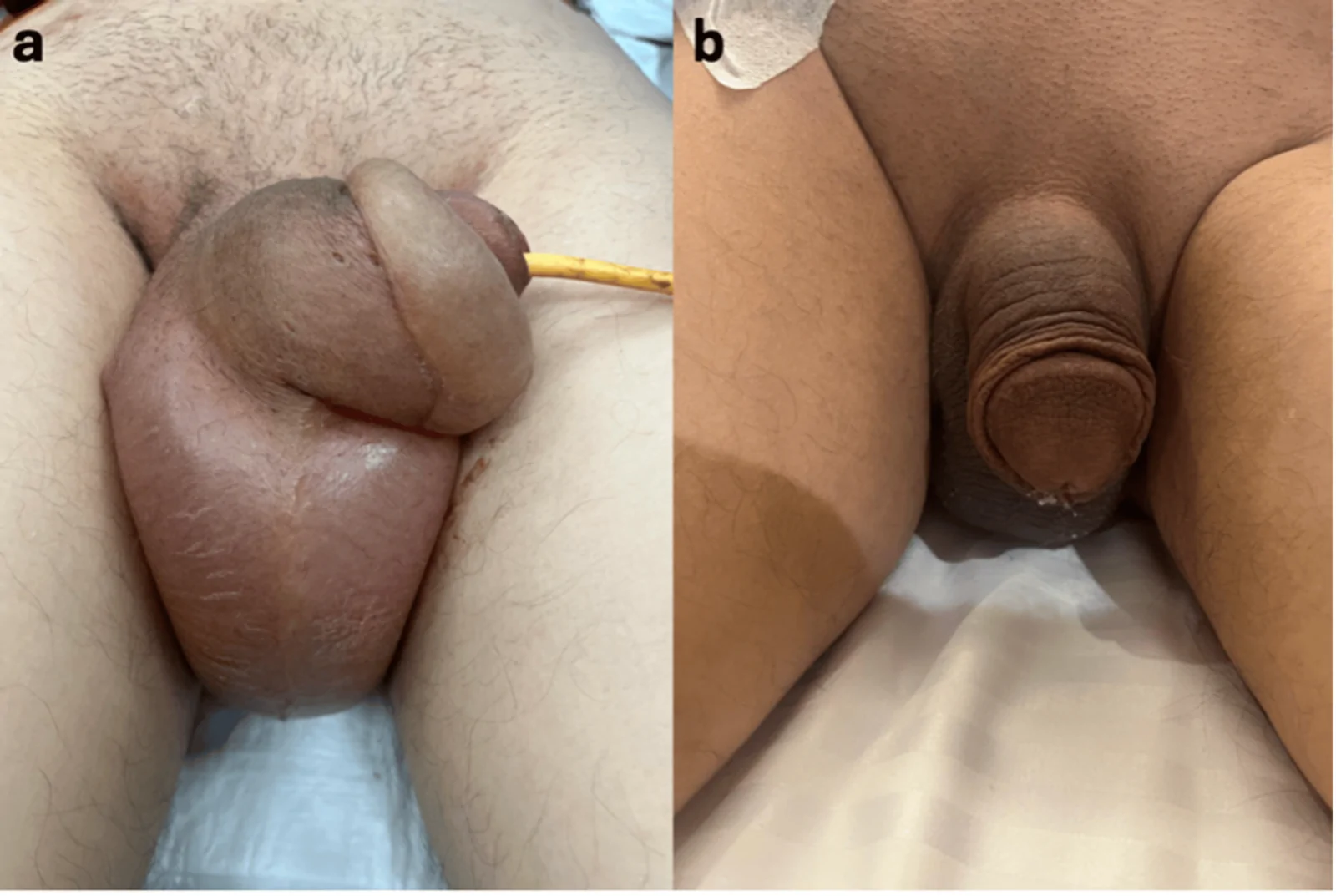
Clinical appearance of scrotal edema before (a) and after (b) surgical treatment of inferior vena cava obstruction. Written informed consent was obtained from the patient for publication of this case report and the accompanying images.

Contrast-enhanced abdominal CT demonstrated a right-sided hilar mass consistent with perihilar cholangiocarcinoma. The patient underwent right hepatectomy through an upper midline incision with a right subcostal extension. Liver transection was completed using the clamp-crush technique with intermittent Pringle maneuvers. Routine thromboprophylaxis with low-molecular-weight heparin (enoxaparin) was started on postoperative day 1 at a daily subcutaneous dose of 0.6 cc. The first postoperative day was uneventful. Liver enzymes increased as expected after major hepatectomy (AST 283 U/L, ALT 222 U/L), whereas coagulation parameters remained acceptable. On postoperative day 2, urine output decreased markedly to 0.45 mL/kg/hour. At the same time, the patient developed abdominal distension, tachycardia, and sudden swelling of the scrotum (Figure 1). 

**Figure 2:**
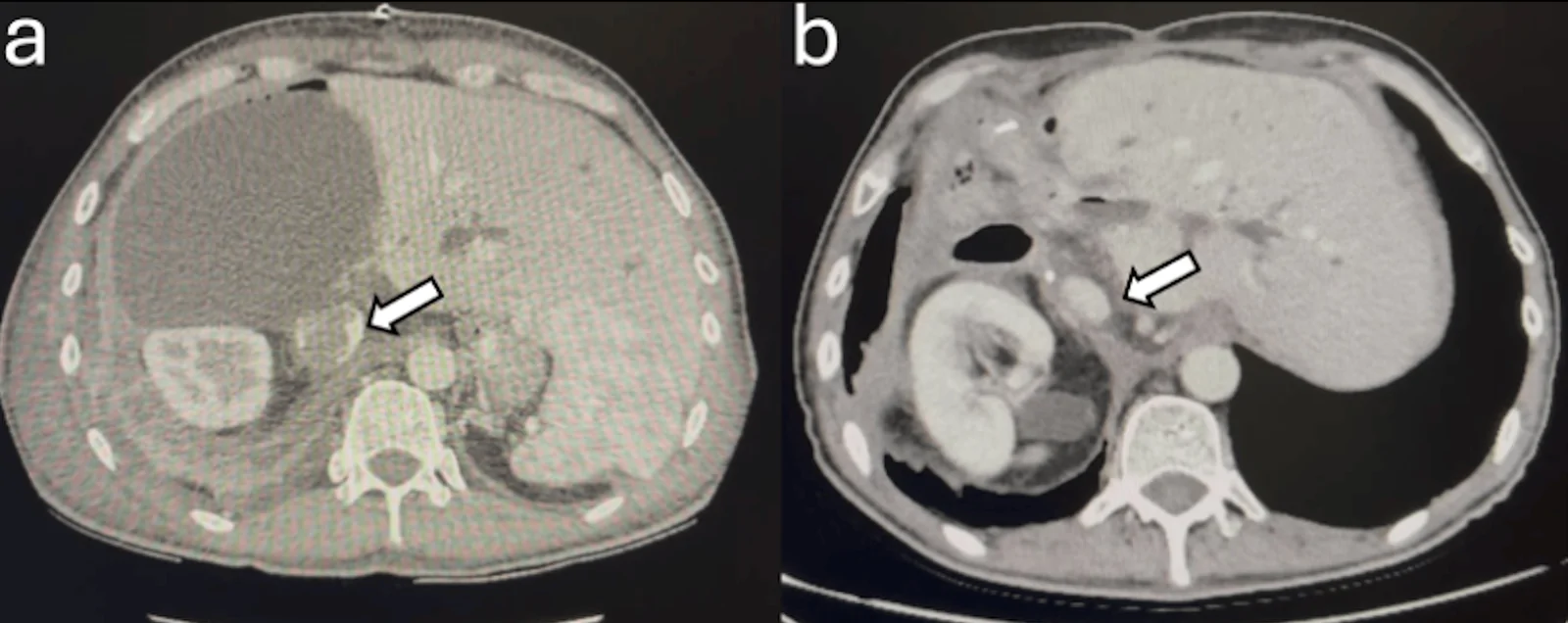
Contrast-enhanced abdominal CT findings.

Serum creatinine increased from 1.13 to 2.84 mg/dL, while the estimated glomerular filtration rate fell from 71.4 to 23.5 mL/min/1.73 m². Blood urea nitrogen also increased to 52.2 mg/dL, and the serum albumin level was 3.0 g/dL. Scrotal Doppler ultrasonography demonstrated preserved bilateral testicular perfusion and diffuse edema of the scrotal soft tissues without evidence of torsion or other intrinsic testicular pathology. Because the rapid deterioration in renal function could not be explained by the clinical findings alone, CT angiography was obtained. The scan demonstrated partial thrombosis of the IVC just below the hepatic venous confluence. The thrombus measured approximately 5 cm in length and resulted in marked narrowing of the caval lumen (Figure 2a). 

The patient was taken back to the operating room because of progressive acute kidney injury and CT findings suggesting mechanical obstruction of the IVC. Exploration demonstrated narrowing of the IVC close to the right hepatic vein stump. After vascular control had been established, the vena cava was opened, and fresh thrombus was removed. The stenotic segment was then enlarged using an autologous patch harvested from the adjacent parietal peritoneum. Restoration of caval flow was confirmed before closure.

Clinical improvement became evident shortly after reoperation. Urine output increased to 3400 mL/24 h during the first postoperative day, and serum creatinine progressively returned to a normal level of 1.10 mg/dL. Before discharge, creatinine was 0.50 mg/dL with normal renal function and coagulation parameters. Postoperative CT demonstrated restoration of IVC patency after thrombectomy and patch repair (Figure 2b). Scrotal edema started to regress within 24 hours after surgery and had resolved completely by day 10 after the initial hepatectomy (Figure 1b). The remainder of the hospital stay was uneventful. Therapeutic anticoagulation with low-molecular-weight heparin was continued throughout hospitalization at a subcutaneous dose of 0.6 mL twice daily. The patient was discharged on the fifth day after reoperation in good clinical condition. At the eight-month follow-up, he remained free of recurrent thrombosis, renal dysfunction, or other vascular complications.

## Discussion

Postoperative IVC thrombosis is an uncommon complication of major liver resection [1]. When it occurs, the diagnosis may be difficult because the initial findings are often nonspecific. Clinical manifestations of IVC thrombosis may include lower-extremity edema, ascites, renal dysfunction, and other signs of venous congestion [4]. In our patient, however, the first remarkable finding was sudden scrotal edema.

The mechanism of thrombosis was probably mechanical rather than thrombotic alone. During reoperation, focal narrowing of the IVC was found close to the right hepatic vein stump. After right hepatectomy, the remaining liver may shift into the empty subphrenic space and change the orientation of the vena cava. Reduced venous flow together with postoperative hypercoagulability probably contributed to thrombus formation [3].

Scrotal edema is usually associated with heart failure, cirrhosis, hypoalbuminemia, or local scrotal disease [5]. It is not generally considered a sign of postoperative caval obstruction. Although hypoalbuminemia may have contributed, the sudden onset of scrotal swelling with oliguria and worsening renal function prompted further investigation. CT demonstrated IVC thrombosis, indicating venous obstruction as the main cause. Doppler ultrasonography excluded testicular torsion and showed preserved testicular perfusion [6].

The differential diagnosis of acute scrotal swelling after abdominal surgery includes testicular torsion, epididymo-orchitis, postoperative hematoma, hydrocele, incarcerated inguinal hernia, Fournier gangrene, generalized edema, and spermatic vein thrombosis. Because these conditions require different management strategies, careful examination and Doppler ultrasonography remain essential. When no local cause is identified and renal function deteriorates simultaneously, CT angiography should be considered to evaluate the IVC [6].

The rapid increase in serum creatinine was consistent with venous congestive nephropathy. Elevated renal venous pressure reduces the filtration gradient despite preserved arterial inflow. The immediate recovery of renal function after thrombectomy strongly suggests that venous obstruction was responsible for the acute kidney injury [7].

Different treatment options have been described for postoperative IVC obstruction, including anticoagulation, endovascular intervention, and open surgical repair [8]. In the present case, imaging and intraoperative findings demonstrated a fixed mechanical stenosis. For this reason, thrombectomy alone would not have been sufficient. Enlargement of the narrowed segment with an autologous peritoneal patch relieved the obstruction and restored venous flow without the need for prosthetic material. Autologous peritoneal patches have been reported to provide a practical, biocompatible, and readily available option for IVC reconstruction in hepatopancreatobiliary surgery [9-11].

Only a few reports have described postoperative IVC thrombosis after hepatectomy [1]. Acute scrotal edema has not been previously reported as the presenting feature of this complication. In our patient, scrotal edema appeared before irreversible organ damage developed and led to early imaging and reoperation. This observation may help clinicians recognize this rare complication at an earlier stage.

## Conclusions

Isolated IVC thrombosis should be considered in patients who develop sudden scrotal edema after major hepatectomy, particularly when it is accompanied by oliguria or worsening renal function. Early CT angiography is useful for confirming the diagnosis. When mechanical stenosis is present, prompt surgical correction may restore venous drainage and prevent permanent renal injury.
